# Characteristics of covid-19 in miners and former miners with a history of silicosis

**DOI:** 10.17843/rpmesp.2025.423.14698

**Published:** 2025-08-20

**Authors:** Luis Guillermo Farmer-Aldunce, Ana María Salazar Bugueño, Marisol Concha-Barrientos, Gustavo Contreras Tudela, Olivia Janett Horna-Campos

**Affiliations:** 1 Escuela de Salud Pública, Facultad de Medicina, Universidad de Chile, Santiago, Chile. Universidad de Chile Escuela de Salud Pública Facultad de Medicina Universidad de Chile Santiago Chile; 2 Instituto de Seguridad del Trabajo (IST), Santiago, Chile. Instituto de Seguridad del Trabajo (IST) Santiago Chile; 3 Clínica Alemana, Valdivia, Chile. Clínica Alemana Valdivia Chile; 4 Clinica Red Salud Mayor Temuco, Temuco, Chile. Clinica Red Salud Mayor Temuco Temuco Chile

**Keywords:** Silicosis, Epidemiology, Complications, COVID-19

## Abstract

During the SARS-CoV-2 pandemic, it was suggested that individuals with chronic lung diseases, such as silicosis, could be at increased risk of complications; however, the evidence remains limited. This study describes the prevalence of COVID-19 among 51 miners and former miners with silicosis who were part of an occupational surveillance program. 96% were over 60 years old, 37.2% had moderate or severe silicosis, and 67% had at least one comorbidity. The prevalence of COVID-19 was 15.7%, lower than the general population, with mild cases and no reported deaths. 94.1% had received at least two doses of vaccine; of these, 12.5% were affected by COVID-19, compared to 66.7% of those who were not vaccinated. These findings suggest that vaccination and preventive measures may have contributed to reduce transmission and severity, highlighting the importance of maintaining protection for individuals with chronic respiratory diseases.

## INTRODUCTION

The pandemic caused by SARS-CoV-2 has subsided, but its impact on the affected populations has been and will likely continue to be very significant. According to the World Health Organization (WHO), more than 770 million people have been affected by the virus, of whom 6.9 million have died. It is also estimated that between 5% and 10% of COVID-19 survivors have developed the syndrome known as “long COVID” or “post-COVID condition,” a set of clinical manifestations that impair the quality of life of affected individuals to varying degrees ^1^.

People with obesity, arterial hypertension, diabetes ^2^^-^^4^, and those affected by chronic respiratory diseases, such as chronic obstructive pulmonary disease (COPD) ^5^^,^^6^ and diffuse interstitial lung disease (DILD) were among the most affected by COVID-19. In this line, a study revealed that those suffering from DILD had a four times higher risk of dying than those without this condition ^7^.

Occupational exposures to silica generate a type of DILD known as silicosis. A review of the health status of mine workers in South Africa identified that living conditions allowed for the rapid spread of tuberculosis, human immunodeficiency virus, and SARS-CoV-2. However, the authors concluded that, up to that point, there was insufficient evidence to consider pneumoconiosis as a risk factor for COVID-19 ^8^. In Chile, it is estimated that around 500,000 workers were exposed to silica during the pandemic, and about 8,000 suffered from silicosis ^9^. The purpose of this study is to characterize COVID-19 in miners and former miners with a history of silicosis in Chile.

**KEY MESSAGES**

Motivation for the study. People with chronic respiratory diseases were the most affected by COVID-19. However, this risk is unclear in people with silicosis.

Main findings. This study shows a lower prevalence of COVID-19 in miners and former miners with a history of silicosis compared to the general population.

Public health implications. These findings reinforce the importance of maintaining surveillance programs and actively promoting vaccination and protective measures in people with chronic respiratory pathologies due to occupational exposure.

## THE STUDY

A cross-sectional study was conducted. The study population consisted of miners and former miners belonging to the National Silicosis Corporation between March 2020 and December 2022. The sampling was non-probabilistic by convenience. All those who voluntarily agreed to participate were included, after being informed and signing the informed consent. All participants answered a structured questionnaire of 40 questions, which collected sociodemographic data, health history, vaccination, and COVID-19 diagnosis. The survey had an average duration of 15 minutes and was conducted at the premises of the National Silicosis Corporation (https://www.cnsilicosis.cl/cns/index.html).

### Variables

The diagnosis of COVID-19 was made with a positive RT-PCR test, during the period from March 2020 to the time of the study (August and December 2022). Covariates considered were age in completed years, signs and symptoms, degree of silicosis (mild, moderate, severe) according to the profusion of opacities as described by the NIOSH guide for reading radiographs with ILO technique, history of comorbidities (yes/no), hypertension (yes/no), diabetes (yes/no), smoking history (yes/no), supplemental oxygen (yes/no), hospitalization (yes/no), number of COVID-19 vaccine doses, and the application of preventive measures (hand washing, mask use, physical distancing, and ventilation) categorized as always, sometimes, and never, during the last month.

### Statistical analysis

A descriptive analysis of the sample was performed, considering sociodemographic, clinical, and occupational characteristics, using percentages, frequencies, means, and standard deviation (SD), according to the measurement scale of the variables. The prevalence of COVID-19 was described, characterizing the cases based on the covariates. The normality of the numerical variables was assessed using the Shapiro-Wilk test. All analyses were performed with Stata version 18.

### Ethical considerations

Informed consent was obtained from all participants. Each questionnaire was identified with a numerical code to maintain confidentiality. This study was conducted with the approval of the Ethics Committee for Research with Human Beings of the Faculty of Medicine of the University of Chile. Project: No. 213-2021 (act file: No. 195).

## FINDINGS

The registry of the National Silicosis Corporation included 88 people. Between 2018 and 2022, 10 deaths were recorded, none attributed to COVID-19. 51 participants were interviewed and their COVID-19 diagnosis was verified, which corresponds to 65.4% of the total. All participants were men. The mean age was 72.1 (SD: 7.6) years, with a minimum and maximum value of 50 and 94 years, respectively (p-value of the Shapiro-Wilk test=0.217).

15.7% had a positive PCR test result for SARS-CoV-2 (95% CI: 7.9−28.7). Among the cases, 62% reported headache and 50% had cough, dyspnea, anosmia, and ageusia. 67% of the participants reported at least one comorbidity; among them, 53% had arterial hypertension and 29.4% had diabetes mellitus. Regarding preventive measures, 80.4% indicated always using a mask in the presence of other people; 88.2% (n=45) declared always maintaining a physical distance of one to two meters; and 90.2% (n=46) stated that they ensured ventilation of spaces, even during winter. In addition, 92.2% (n=47) reported practicing hand hygiene after contact with objects from outside the home or after physical interaction with other people.

94.1% (n=48) of the interviewees had a complete vaccination schedule against SARS-CoV-2 (two or more doses); among them, only 12.5% (n=6) fell ill with COVID-19, in contrast to 66.7% among the unvaccinated (n=3). Although 23.5% (n=12) reported using supplemental oxygen by medical prescription, only two of them presented with COVID-19 (Table 1).

**Table 1:** Sociodemographic, clinical, and occupational characteristics of patients with silicosis according to COVID-19 diagnosis

Variables			COVID-19		Total=51 n (%)
			No=43	Yes=8	
			n (%)	n (%)	
Age					
	Mean (DE)		72,7 (DE 7,4)	68,7 (8,5)	72,1 (7,6)
Degree of silicosis					
	Mild		28 (87,5)	4 (12,5)	32 (62,8)
	Moderate-severe		15 (79,0)	4 (21,0)	19 (37,2)
Hospitalization					
	No		0 (0,0)	6 (100,0)	6 (11,8)
	Yes		0 (0,0)	2 (100,0)	2 (3,9)
Supplemental oxygen					
	No		33 (84,6)	6 (15,4)	39 (76,5)
	Yes		10 (83,3)	2 (16,7)	12 (23,5)
Vaccine doses					
	0		1 (33,3)	2 (66,7)	3 (5,9)
	≤2		8 (88,9)	1 (11,1)	9 (17,6)
	≥3		34 (87,2)	5 (12,8)	39 (76,5)
Smoking history					
	No		36 (83,7)	7 (16,3)	43 (84,3)
	Yes		7 (87,5)	1 (12,5)	8 (15,7)
Comorbidities					
	No		13 (76,5)	4 (23,5)	17 (33,3)
	Yes		30 (88,2)	4 (11,8)	34 (66,7)
		AHT^b^	15 (83,3)	3 (16,7)	18 (53,0)^a^
		Diabetes mellitus^b^	8 (80,0)	2 (20,0)	10 (29,4)^a^
Preventive measures					
	Do you wear a mask in the presence of other people?				
		Always	34 (82,9)	7 (17,1)	41 (80,4)
		Sometimes	9 (88,9)	1 (11,1)	10 (19,6)
		Never	0 (0,0)	0 (0,0)	0 (0,0)
	Do you maintain physical distance from other people?				
		Always	38 (88,4)	7 (15,6)	45 (88,2)
		Sometimes	5 (83,3)	1 (16,7)	6 (11,8)
		Never	0 (0,0)	0 (0,0)	0 (0,0)
	Do you ventilate closed spaces?				
		Always	39 (84,8)	7 (15,2)	46 (90,2)
		Sometimes	3 (75,0)	1 (25,0)	4 (7,8)
		Never	1 (100,0)	0 (0,0)	1 (2,0)
	How often do you wash your hands?				
		Always	39 (84,8)	7 (15,2)	47 (92,2)
		Sometimes	4 (100,0)	0 (0,0)	4 (7,8)
		Never	0 (0,0)	0 (0,0)	0 (0,0)

a Percentage in relation to the people who presented at least one comorbidity.

b Only the comorbidities with the highest reporting are described.

AHT: arterial hypertension, SD: standard deviation.

## DISCUSSION

It was found that the prevalence of COVID-19 infection was 15.7% (n=8) in a context of broad compliance with preventive measures.

The prevalence of COVID-19 observed in this study was even lower than the 21.7% reported for the general population as of July 31, 2022 ^10^. Scientific studies suggest that patients with pneumoconiosis are more likely to get sick from viral, bacterial, and/or fungal infections. The finding of a similar percentage of COVID-19 cases among patients with silicosis, at different stages of the disease, in this study sample, compared to the general population, could be associated with the preventive measures correctly adopted by these patients.

Since the beginning of the pandemic, it was assumed that patients with chronic lung diseases, such as silicosis, were particularly vulnerable to SARS-CoV-2 infection, due to their general condition and possible immunological alterations. This was supported by evidence that patients with silicosis have a three times higher risk of developing tuberculosis, as well as an increased risk of lung cancer ^11^. Likewise, it was thought that occupational factors associated with silica exposure could increase both the severity of the infection and the post-COVID-19 sequelae. However, no signs of this were observed in this study. In fact, research conducted in a cohort of miners in India also found no conclusive relationship between occupational exposure to silica and the incidence of COVID-19 ^12^.

The high percentage of compliance with preventive measures, related to the condition of being chronically ill, may introduce a bias in this study, as the recognition of silicosis as a higher-risk pathology may have motivated the adoption of stricter preventive measures ^13^. In this sense, it is expected that patients with silicosis would have a lower incidence of COVID-19, as has been observed in this study. In fact, the study by Gomero-Cuadra et al. showed that there was a lower probability of contagion among construction workers who worked in person than among those who worked remotely ^14^.

Regarding the SARS-CoV-2 vaccine, almost all had a complete vaccination schedule (94%). In this sense, it has been observed that the presence of antibody titers correlates with protection against symptomatic infection over time ^15^. However, the effectiveness of vaccines has varied, partly due to the progressive decrease in antibody titers, which reduces the ability to prevent infection. In addition, the emergence of new viral variants has modified both the immune response and the virulence and reproductive capacity of the virus. In this context, the administration of two or more doses could have contributed to maintaining the effectiveness of the vaccine and providing greater protection against the different variants over time ^15^^,^^16^, even in populations with chronic respiratory diseases ^16^.

In this study, although 96% of the sample was over 60 years old, 37.2% had moderate or severe silicosis, and 67% had at least one other concomitant disease. Most of the COVID-19 cases were mild and only two required hospitalization, showing good evolution without requiring intensive care procedures. The clinical manifestations did not differ from what was observed in the general population, mainly highlighting the presence of headache, cough, dyspnea, ageusia, anosmia, and fever ^10^. No deaths or cases of post-COVID-19 were recorded, despite the fact that 53% had a history of arterial hypertension and 29.4% had diabetes mellitus. A study has suggested that, in patients with silicosis, there could be biological mechanisms that would protect them from SARS-CoV-2, possibly related to the extensive pulmonary fibrosis they present, in which chronic inflammation with activated macrophages and lymphocytes is observed ^12^.

Age proved to be a relevant factor, as the patients who presented with COVID-19 were, on average, younger than those without infection. A plausible hypothesis is that younger patients suffered less functional limitation and, therefore, had a more independent and social participation, which exposed them to a higher risk. Likewise, pandemic fatigue in this group may have affected sustained adherence to preventive measures, which decreased their impact on preventing contagion.

One of the main limitations was the sample size. Although no rejections were recorded, it was not possible to contact 30% of the sample due to mobility restrictions during the COVID-19 pandemic. Another important limitation was that the sample was composed of former workers of a state-owned company with its own medical service, so all were under therapeutic surveillance and regular medical control, which implies particular characteristics that limit the generalization of the results to other populations with silicosis. In this context, it is reasonable to assume that a person with silicosis who receives continuous medical care, follows an appropriate treatment, and maintains sustained preventive measures could have an equal or even lower risk of infection than the general population. In addition, structured access to occupational health services may represent an advantage for the prevention, early diagnosis, and timely management of diseases in this population ^17^.

In conclusion, in this series of patients with silicosis, a low infection rate by COVID-19 was observed with no severe cases or deaths. This result could be explained by a high adherence to preventive measures and the high vaccination coverage with complete schedules in most of the participants. Consequently, health services should actively encourage vaccination in populations with underlying chronic pathologies, not only against SARS-CoV-2, but also against other transmissible respiratory diseases, such as influenza and pneumococcus, which can exacerbate or worsen the health status. The findings suggest that active surveillance programs, by facilitating clinical follow-up and adherence to prevention strategies, can play a relevant role in mitigating the risk of transmissible diseases in vulnerable groups. The need to maintain surveillance programs and to actively promote vaccination and protective measures in people with chronic respiratory pathologies is highlighted.
