# Supplementary material for: Characteristics of covid-19 in miners and former miners with a history of silicosis
Source: Rev Peru Med Exp Salud Publica. 2025 Aug 20;42(3):318–22. doi: 10.17843/rpmesp.2025.423.14698 (PMC12679966; doi:10.17843/rpmesp.2025.423.14698)
Supplement: Translation

# Translation: Características de la COVID-19 en mineros y exmineros con antecedentes de silicosis

**Authors:** Luis Guillermo Farmer-Aldunce, Ana María Salazar Bugueño, Marisol Concha-Barrientos, Gustavo Contreras Tudela, Olivia Janett Horna-Campos

**Affiliations:** 1 Escuela de Salud Pública, Facultad de Medicina, Universidad de Chile, Santiago, Chile.; 2 Instituto de Seguridad del Trabajo (IST), Santiago, Chile.; 3 Clínica Alemana, Valdivia, Chile.; 4 Clínica RedSalud Mayor Temuco, región, Chile.

**Keywords:** Silicosis, Epidemiología, Complicaciones, COVID-19

## Abstract

Durante la pandemia por SARS-CoV-2 se planteó que las personas con enfermedades pulmonares crónicas, como la silicosis, podrían tener mayor riesgo de complicaciones; sin embargo, la evidencia es limitada. Este estudio describe la prevalencia de COVID-19 en 51 mineros y exmineros con silicosis que formaban parte de un programa de vigilancia ocupacional. El 96% tenía más de 60 años, el 37,2% presentaba silicosis moderada o grave y el 67% padecía al menos una comorbilidad. La prevalencia de COVID-19 fue del 15,7%, menor que en la población general, con casos leves y sin fallecimientos. El 94,1 % contaba con al menos dos dosis de vacuna; de estos, el 12,5% presentó COVID-19, frente al 66,7% de los no vacunados. Estos hallazgos sugieren que la vacunación y las medidas preventivas pudieron haber contribuido a reducir la transmisión y la gravedad, destacando la importancia de mantener la protección de personas con enfermedades respiratorias crónicas.

## INTRODUCCIÓN

La pandemia provocada por el SARS-CoV-2 ha remitido, pero su impacto en las poblaciones afectadas ha sido y probablemente seguirá siendo muy significativo. Según la Organización Mundial de la Salud (OMS), más de 770 millones de personas se han visto afectadas por el virus, de las cuales 6,9 millones han fallecido. Se estima, además, que entre el 5% y el 10% de los supervivientes de COVID-19 han desarrollado el síndrome conocido como «COVID persistente» o «condición pos-COVID», un conjunto de manifestaciones clínicas que deterioran la calidad de vida de las personas afectadas en distintos grados ^1^.

Las personas con obesidad, hipertensión arterial, diabetes ^2^^-^^4^, y afectadas por enfermedades respiratorias crónicas, como la enfermedad pulmonar obstructiva crónica (EPOC) ^5^^,^^6^ y la enfermedad pulmonar intersticial difusa (EPID) estuvieron entre las más afectadas por la COVID-19. En esta línea, un estudio reveló que quienes padecían EPID tenían un riesgo de morir cuatro veces mayor que quienes no presentaban esta condición ^(^^7^.

Las exposiciones ocupacionales a la sílice generan un tipo de EPID conocida como silicosis. Una revisión sobre el estado de salud de trabajadores mineros en Sudáfrica identificó que las condiciones de vida permitieron la rápida propagación de la tuberculosis, el virus de inmunodeficiencia humana y el SARS-CoV-2. No obstante, los autores concluyeron que, hasta ese momento, no existía evidencia suficiente para considerar la neumoconiosis como un factor de riesgo para la COVID-19 ^8^. En Chile, se estima que alrededor de 500 000 trabajadores estuvieron expuestos a sílice durante la pandemia y alrededor de 8000 padecían silicosis ^9^. El propósito de este estudio es caracterizar la COVID-19 en mineros y exmineros con antecedentes de silicosis en Chile.

**MENSAJE CLAVE**

Motivación para realizar el estudio. Las personas con enfermedades respiratorias crónicas fueron las más afectadas por la COVID-19. Sin embargo, este riesgo no está claro en las personas con silicosis.

Principales hallazgos. Este estudio muestra una menor prevalencia de COVID-19 en mineros y exmineros con antecedentes de silicosis en comparación con la población general.

Implicancias en salud pública. Estos hallazgos refuerzan la importancia de mantener programas de vigilancia y de promover activamente la vacunación y las medidas de protección en personas con patologías respiratorias crónicas por exposición laboral.

## EL ESTUDIO

Se realizó un estudio transversal. La población de estudio fueron mineros y exmineros pertenecientes a la Corporación Nacional de Silicosis durante marzo del 2020 y diciembre del 2022. El muestreo fue no probabilístico por conveniencia. Se incluyó a todos los que aceptaron participar voluntariamente, previa información y firma del consentimiento informado.

Todos los participantes respondieron un cuestionario estructurado de 40 preguntas, que recolectó datos sociodemográficos, antecedentes de salud, vacunación y diagnóstico de la COVID-19. La encuesta tuvo una duración promedio de 15 minutos y se realizó en las dependencias de la Corporación Nacional de la Silicosis (https://www.cnsilicosis.cl/cns/index.html).

### Variables

El diagnóstico de la COVID-19 se realizó con la prueba RT-PCR positiva, durante el periodo comprendido desde marzo del 2020 hasta el momento del estudio (agosto y diciembre del 2022). Como covariables se consideró la edad en años cumplidos, signos y síntomas, grado de silicosis (leve, moderada severa) según la profusión de las opacidades por descripción de la guía de la NIOSH para la lectura de radiografías con técnica OIT, antecedente de comorbilidades (sí/no), hipertensión (sí/no), diabetes (sí/no), historia de tabaco (sí/no), oxígeno suplementario (sí/no), hospitalización (sí/no), número de dosis de vacuna COVID-19 y la aplicación de medidas preventivas (lavado de manos, uso de mascarilla, distancia física y ventilación) categorizados como siempre, a veces y nunca, durante el último mes.

### Análisis estadístico

Se realizó un análisis descriptivo de la muestra, considerando las características sociodemográficas, clínicas y laborales, utilizando porcentajes, frecuencias, medias y desviación estándar (DE), según la escala de medida de las variables. Se describió la prevalencia de la COVID-19, caracterizando los casos con base en las covariables. La normalidad de las variables numéricas se evaluó mediante la prueba de Shapiro Wilk. Todos los análisis se realizaron con Stata versión 18.

### Consideraciones éticas

Se obtuvo el consentimiento informado de todos los participantes. Cada cuestionario se identificó con un código numérico para mantener la confidencialidad. Este estudio se realizó con la aprobación del Comité de Ética en Investigación con Seres Humanos de la Facultad de Medicina de la Universidad de Chile. Proyecto: N.º 213-2021(archivo acta: N.º 195).

## HALLAZGOS

El registro de la Corporación Nacional de Silicosis incluía 88 personas. Entre el 2018 y 2022, se registraron 10 fallecimientos, ninguna atribuida a la COVID-19. Se entrevistó a 51 participantes y se verificó su diagnóstico de COVID-19, lo que corresponde al 65,4% del total. Todos los participantes fueron hombres. La media de la edad fue de 72,1 (DE: 7,6) años, con un valor mínimo y máximo de 50 y 94 años, respectivamente (valor de p de la prueba de Shapiro-Wilk=0,217).

El 15,7% tuvo un resultado positivo en la prueba PCR para SARS-CoV-2 (IC 95%: 7,9−28,7). Entre los casos, el 62% refirió cefalea y el 50% presentó tos, disnea, anosmia y ageusia. El 67% de los participantes reportó al menos una comorbilidad; entre ellos, el 53% tenía hipertensión arterial y el 29,4% diabetes mellitus. Respecto de las medidas preventivas, el 80,4% indicó utilizar siempre la mascarilla en presencia de otras personas; el 88,2% (n=45) declaró mantener siempre una distancia física de uno a dos metros; y el 90,2% (n=46) afirmó procurar la ventilación de los espacios, incluso durante el invierno. Además, el 92,2% (n=47) manifestó realizar higiene de manos tras el contacto con objetos provenientes fuera de casa o luego de interactuar físicamente con otras personas.

El 94,1% (n=48) de los entrevistados contaba con esquema completo de vacunación contra el SARS-CoV-2 (dos o más dosis); entre ellos, sólo el 12,5% (n=6) enfermó de COVID-19, en contraste con el 66,7% entre los no vacunados (n=3). Aunque el 23,5% (n=12) refirió uso de oxígeno suplementario por indicación médica, solo dos de ellos presentaron COVID-19 (tabla 1).

**Tabla 1:** Características sociodemográficas, clínicas y laborales de los pacientes con silicosis según diagnóstico de la COVID-19.

Variables			COVID-19		Total=51 n (%)
			No=43 n (%)	Sí=8 n (%)	
Edad					
	Media (DE)		72,7 (7,4)	68,7 (8,5)	72,1 (7,6)
Grado de silicosis					
	Leve		28 (87,5)	4 (12,5)	32 (62,8)
	Moderado-severo		15 (79,0)	4 (21,0)	19 (37,2)
Hospitalización					
	No		0 (0,0)	6 (100,0)	6 (11,8)
	Sí		0 (0,0)	2 (100,0)	2 (3,9)
Oxígeno suplementario					
	No		33 (84,6)	6 (15,4)	39 (76,5)
	Sí		10 (83,3)	2 (16,7)	12 (23,5)
Dosis de vacuna					
	0		1 (33,3)	2 (66,7)	3 (5,9)
	≤2		8 (88,9)	1 (11,1)	9 (17,6)
	≥3		34 (87,2)	5 (12,8)	39 (76,5)
Historia de tabaco					
	No		36 (83,7)	7 (16,3)	43 (84,3)
	Sí		7 (87,5)	1 (12,5)	8 (15,7)
Comorbilidades					
	No		13 (76,5)	4 (23,5)	17 (33,3)
	Sí		30 (88,2)	4 (11,8)	34 (66,7)
	HTA^b^		15 (83,3)	3 (16,7)	18 (53,0)^a^
	Diabetes *mellitus* ^b^		8 (80,0)	2 (20,0)	10 (29,4)^a^
Medidas preventivas					
	¿Usa mascarilla en presencia de otras personas?				
		Siempre	34 (82,9)	7 (17,1)	41 (80,4)
		A veces	9 (88,9)	1 (11,1)	10 (19,6)
		Nunca	0 (0,0)	0 (0,0)	0 (0,0)
	¿Mantiene distancia física respecto a otras personas?				
		Siempre	38 (88,4)	7 (15,6)	45 (88,2)
		A veces	5 (83,3)	1 (16,7)	6 (11,8)
		Nunca	0 (0,0)	0 (0,0)	0 (0,0)
	¿Ventila los lugares cerrados?				
		Siempre	39 (84,8)	7 (15,2)	46 (90,2)
		A veces	3 (75,0)	1 (25,0)	4 (7,8)
		Nunca	1 (100,0)	0 (0,0)	1 (2,0)
	¿Con qué frecuencia lava sus manos?				
		Siempre	39 (84,8)	7 (15,2)	47 (92,2)
		A veces	4 (100,0)	0 (0,0)	4 (7,8)
		Nunca	0 (0,0)	0 (0,0)	0 (0,0)

a Porcentaje en relación con las personas que presentaron al menos una comorbilidad.

b Solo se describen las comorbilidades que tuvieron mayor reporte.

HTA: hipertensión arterial, DE: desviación estándar.

## DISCUSIÓN

Se halló que la prevalencia de infección por la COVID-19 fue del 15,7% (n=8) en un contexto de amplio cumplimiento de medidas preventivas.

La prevalencia de COVID-19 observada en este estudio fue incluso inferior al 21,7% reportado para la población general al 31 de julio de 2022 ^10^. Estudios científicos sugieren que los pacientes portadores de neumoconiosis tienen mayor probabilidad de enfermar por infecciones virales, bacterianas y/o micóticas. El hallazgo de un porcentaje similar de casos de COVID-19 entre los pacientes con silicosis, en distintas etapas de la enfermedad, en esta muestra de estudio, en comparación con la población general, podría estar asociado a las medidas preventivas correctamente adoptadas por estos pacientes.

Desde el inicio de la pandemia, se asumió que los pacientes con enfermedades pulmonares crónicas, como la silicosis, eran particularmente vulnerables a la infección por SARS-CoV-2, debido a su estado general y posibles alteraciones inmunológicas. Esto se sustentaba en la evidencia de que los pacientes con silicosis presentan un riesgo tres veces mayor de desarrollar tuberculosis, así como un riesgo incrementado de cáncer de pulmón ^(^^11^. Asimismo, se pensó que los factores laborales asociados a la exposición a sílice podrían incrementar tanto la gravedad de la infección como las secuelas pos-COVID-19. No obstante, en este estudio no se observaron indicios de ello. De hecho, una investigación realizada en una cohorte de mineros en la India tampoco encontró una relación concluyente entre la exposición ocupacional a sílice y la incidencia de COVID-19 ^12^.

El alto porcentaje de cumplimiento de las medidas preventivas, relacionado con la condición de enfermo crónico, puede introducir un sesgo en este estudio, ya que el reconocimiento de la silicosis como una patología de mayor riesgo pudo haber motivado la adopción de medidas preventivas más estrictas ^13^. En este sentido, es esperable que los pacientes con silicosis presenten menor incidencia de COVID-19, como se ha observado en este estudio. De hecho, el estudio realizado por Gomero-Cuadra *et al*. mostró que existió menor probabilidad de contagio entre los trabajadores de la construcción que se desempeñaron presencialmente que entre aquellos que trabajaron de manera remota ^14^.

En relación con la vacuna contra el SARS-CoV-2, prácticamente todos acreditaron esquema completo de vacunación (94%). En este sentido, se ha observado que la presencia de títulos de anticuerpos se correlaciona con la protección frente a la infección sintomática a lo largo del tiempo ^15^. Sin embargo, la efectividad de las vacunas ha variado, en parte debido a la disminución progresiva de los títulos de anticuerpos, lo que reduce la capacidad de prevención de la infección. Además, la aparición de nuevas variantes virales ha modificado tanto la respuesta inmune como la virulencia y la capacidad reproductiva del virus. En este contexto, la administración de dos o más dosis podría haber contribuido a mantener la efectividad de la vacuna y proporcionar una mayor protección frente a las distintas variantes a lo largo del tiempo ^(^^15^^,^^16^, incluso en poblaciones con enfermedades respiratorias crónicas ^16^.

En este estudio, aunque el 96% de la muestra tenía más de 60 años, el 37,2% tenía silicosis moderada o grave y el 67% tenía al menos otra enfermedad concomitante. La mayoría de los casos de COVID-19 fueron leves y sólo dos requirieron hospitalización, presentando buena evolución sin requerir procedimientos de cuidados intensivos. Las manifestaciones clínicas no diferían de lo observado en la población general, destacándose principalmente la presencia de cefalea, tos, disnea, ageusia, anosmia y fiebre ^10^. No se registraron muertes ni casos de pos-COVID-19, a pesar de que el 53% presentaba antecedentes de hipertensión arterial y el 29,4% de diabetes *mellitus*. Un estudio ha sugerido que, en pacientes con silicosis, podrían existir mecanismos biológicos que los protegerían del SARS-CoV-2, posiblemente relacionados con la extensa fibrosis pulmonar que estos presentan, en la cual se observa una inflamación crónica con macrófagos y linfocitos activados ^12^.

La edad resultó ser un factor relevante, ya que los pacientes que presentaron la COVID-19 eran en promedio más jóvenes que aquellos sin infección. Una hipótesis plausible es que los pacientes más jóvenes sufrían menos limitación funcional y, por lo tanto, tenían una participación más independiente y sociable, lo que los expuso a un mayor riesgo. Asimismo, la fatiga pandémica en este grupo pudo haber afectado la adherencia sostenida a las medidas preventivas, lo que disminuyó su impacto en la prevención del contagio.

Una de las principales limitaciones fue el tamaño de la muestra. Si bien, no se registraron rechazos, no fue posible contactar al 30% de la muestra debido a restricciones de movilidad durante la pandemia de la COVID-19. Otra limitación importante fue que la muestra estuvo compuesta por pacientes ex trabajadores de una empresa estatal con servicio médico propio, por lo que todos se encontraban bajo vigilancia terapéutica y de control médico regular, lo que implica características particulares que limitan la generalización de los resultados a otras poblaciones con silicosis. En este contexto, es razonable suponer que una persona con silicosis que recibe atención médica continua, sigue un tratamiento adecuado y mantiene medidas preventivas sostenidas podría tener un riesgo igual o incluso menor de infección que la población general. Además, el acceso estructurado a servicios de salud ocupacional puede representar una ventaja para la prevención, el diagnóstico temprano y el manejo oportuno de enfermedades en esta población ^17^.

En conclusión, en esta serie de pacientes con silicosis se observó una baja infección por la COVID-19 sin casos graves ni fallecimientos. Este resultado podría explicarse por una alta adherencia a las medidas preventivas y la elevada cobertura de vacunación con esquemas completos en la mayoría de los participantes. En consecuencia, los servicios de salud deben alentar activamente la vacunación en poblaciones con patologías crónicas subyacentes, no solo contra el SARS-CoV-2, sino también contra otras enfermedades transmisibles respiratorias, tales como la influenza y el neumococo, que pueden exacerbar o agravar el estado de salud. Los hallazgos sugieren que los programas de vigilancia activa, al facilitar el seguimiento clínico y la adherencia a las estrategias de prevención, pueden desempeñar un rol relevante en la mitigación del riesgo de enfermedades transmisibles en grupos vulnerables. Se destaca, la necesidad de mantener programas de vigilancia y de promover activamente la vacunación y las medidas de protección en personas con patologías respiratorias crónicas.
